# Quantitative Analyses of Circadian Gene Expression in Mammalian Cell Cultures

**DOI:** 10.1371/journal.pcbi.0020136

**Published:** 2006-10-13

**Authors:** Mariko Izumo, Takashi R Sato, Martin Straume, Carl Hirschie Johnson

**Affiliations:** 1 Department of Biological Sciences, Vanderbilt University, Nashville, Tennessee, United States of America; 2 Cold Spring Harbor Laboratory, Cold Spring Harbor, New York, United States of America; 3 Customized Online Biomathematical Research Applications, Charlottesville, Virginia, United States of America; University College London, United Kingdom

## Abstract

The central circadian pacemaker is located in the hypothalamus of mammals, but essentially the same oscillating system operates in peripheral tissues and even in immortalized cell lines. Using luciferase reporters that allow automated monitoring of circadian gene expression in mammalian fibroblasts, we report the collection and analysis of precise rhythmic data from these cells. We use these methods to analyze signaling pathways of peripheral tissues by studying the responses of Rat-1 fibroblasts to ten different compounds. To quantify these rhythms, which show significant variation and large non-stationarities (damping and baseline drifting), we developed a new fast Fourier transform–nonlinear least squares analysis procedure that specifically optimizes the quantification of amplitude for circadian rhythm data. This enhanced analysis method successfully distinguishes among the ten signaling compounds for their rhythm-inducing properties. We pursued detailed analyses of the responses to two of these compounds that induced the highest amplitude rhythms in fibroblasts, forskolin (an activator of adenylyl cyclase), and dexamethasone (an agonist of glucocorticoid receptors). Our quantitative analyses clearly indicate that the synchronization mechanisms by the cAMP and glucocorticoid pathways are different, implying that actions of different genes stimulated by these pathways lead to distinctive programs of circadian synchronization.

## Synopsis

The circadian biological clock controls the adaptation of animals and plants to the daily environmental cycle of light and darkness. As such, this clock is responsible for jet lag and has consequences for mental health (e.g., depression), physical health (e.g., athletic performance and the timing of heart attacks), and social issues (e.g., shift work). The central circadian pacemaker is located in the hypothalamus of the mammalian brain, but essentially the same oscillating system operates in nonneural tissues. Using luciferase, an enzyme that emits light, the authors could monitor circadian gene expression in mammalian fibroblasts via luminescence emission that is controlled by the biological clock. Using this method, they report the collection and analysis of precise rhythmic data from these cells. These methods were used to analyze signaling pathways by studying the responses of fibroblasts to a variety of different treatments, including drugs, growth factors, and serum. The authors developed a new analysis procedure that specifically optimizes the quantification of amplitude for cyclic data to analyze these rhythms. This enhanced analysis method successfully distinguishes among the various signaling treatments for their rhythm inducing properties. The quantitative analyses clearly indicate that the synchronization mechanisms by the cyclic AMP and glucocorticoid pathways are different. Therefore, these pathways lead to distinctive programs of circadian synchronization.

## Introduction

Among temporally regulated processes, the circadian clock is unique in that it operates precisely on a cycle of approximately 24 h to regulate time-dependent processes such as sleep–wake cycles and body temperature fluctuations. Molecular components of the mammalian circadian clock have been identified and cloned [1]. They constitute at least two interlocked negative feedback loops: one loop is composed of PER1/PER2 and CRY1/CRY2 as repressing factors that inhibit their own transcription, and CLOCK/BMAL1 transcription factors as positive elements to activate clock-controlled genes from E-boxes on genetic regulatory elements; the other loop is an interlocked circuit in which REV-ERBα and RORα regulate *bmal1* transcription [2]. The core clock components have been extensively studied, but many questions remain: (1) how circadian oscillators are entrained; (2) how the individual oscillators that are present throughout the body are coordinated; and (3) what molecular mechanisms underlie these resetting and synchronization events.

While it might be simplistically thought that the “master” oscillator in the suprachiasmatic nucleus (SCN) of the hypothalamus could govern the rhythmic expression of peripheral tissues by driving overt rhythms in physiology and behavior, data from mammals, *Drosophila,* and other organisms have long suggested the existence of multi-oscillators that were organized in a hierarchical fashion [3,4]. Studies showing that isolated peripheral tissues from *Drosophila* [5], zebrafish [6], rats [4], and even immortalized mammalian cultured cells such as Rat-1 fibroblasts [7] and NIH3T3 cells [8] are capable of generating circadian rhythmicity of gene expression in vitro have provided experimental evidence for hierarchically organized multi-oscillators. In addition to the theoretical concept of oscillators being expressed throughout the organism, the fact that tissue culture cells contain autonomous functional clocks has practical significance. For example, whereas SCN tissue is difficult to obtain and manipulate, cell cultures have important advantages: they are easy to maintain, accessible to molecular genetic tools, and can produce the large amount of material that is necessary for biochemical assays. Because of these advantages, cultured cells have provided an excellent alternative to the SCN for the study of the molecular and biochemical mechanisms of mammalian circadian systems in vitro [8–20].

To facilitate the collection of highly time-resolved data from cell cultures, we and others developed an in vitro bioluminescence reporter system to study the dynamics of temporal expression of clock genes in mammals [13,15,20–23]. This noninvasive automated monitoring system is a powerful tool to study circadian clocks in mammals. However, quantitative analysis of circadian rhythms in peripheral tissues and cultured cells is challenging for a variety of reasons. First, the response of cell cultures to different treatments (e.g., drugs, hormones) is highly variable. Second, the rhythms of the cell cultures exhibit damping (i.e. variance non-stationarities; see [4,15]). Third, these rhythms often show unstable baseline shifting (i.e., mean non-stationarities) that changes from experiment to experiment, or even from sample to sample. In the context of studying input signaling pathways, it is particularly challenging to statistically differentiate between residual low-amplitude rhythms (such as those stimulated by solvent controls or media changes) and high-amplitude rhythms that have been induced by drugs/hormones of interest. Thus, we set out to develop a computational method to analyze the data from cell culture experiments for (1) the presence of rhythmicity, (2) the significance level of the rhythmicity, and (3) the “strength” of the resulting oscillation.

We used fast Fourier transform–nonlinear least squares (FFT-NLLS) analysis [24,25] coupled with detrending to remove mean non-stationarities and variance non-stationarities that are consistently encountered in the Rat-1 cell data. Analysis in this manner permits assignment of values to parameters characterizing the period and phase as well as the degree of rhythmicity via relative amplitude error (RAE). To quantitatively assess oscillatory “strength,” we developed a method for analyzing the *amplitude* of the rhythms. Amplitude analyses have historically been avoided as gauges of circadian pacemakers because the amplitude of an output rhythm will be a reflection of not only the pacemaker's amplitude, but also of the output pathway characteristics, including transients, masking, and other amplitude effects [26]. The importance of amplitude has been appreciated in the modeling of circadian oscillations [27,28] and its role in photoperiodic induction [29], but for physiological and molecular studies, phase and period have traditionally been considered to be the most reliable indicators of pacemaker action to avoid complications of the observed output assay. In particular, by virtue of elegant “two-pulse phase response curve” analyses [3,30], phase-shifting properties were determined to be accurate indicators of the underlying oscillator that were not altered by output considerations. However, our current understanding of the circadian clockwork has advanced to the stage where reasonable molecular candidates that could act as state variables have been identified. We herein describe reporters of promoter activity for the clock genes *mper1, mper2,* and *bmal1;* the transcription rates of these genes are state variables in current transcription/translation feedback models of the mammalian clock [31]. Therefore, quantifying the amplitude of the luminescence rhythms expressed by these reporters as a gauge of the amplitude of the underlying oscillator may contribute important information at a finer level of detail than previously possible.

Our primary goal in the current study lies in elucidating the molecular mechanisms of input signaling and cellular synchronization in the mammalian circadian clock. To examine the precise expression patterns induced by various drug/hormone treatments, we enlisted new luminescence reporters using the *mper2, bmal1,* and *dbp* promoters, thereby extending beyond the repertoire of previous reporter systems [15]. Among ten drug/hormone treatments, the enhanced FFT-NLLS analysis identified (1) 50% horse serum, (2) dexamethasone (Dex), (3) forskolin (Fsk), and (4) epidermal growth factor (EGF) as being most effective in generating high-amplitude rhythms in Rat-1 cells. The association of signaling strength, promoter activation, and the initiation of rhythmicity was further examined in the cases of Fsk and Dex. Our quantitative analyses revealed that the mode of rhythm initiation by Fsk is dramatically different from that by Dex, and this difference is likely to be due to different molecular communication pathways.

## Results

### Modified Luciferase Reporter System in Rat-1 Cells

We previously reported the generation of a stable Rat-1 reporter cell line harboring the firefly luciferase cDNA (Luc) driven by a 3.0-kb *mper1* promoter [15]. While this reporter cell line has been useful in examining rhythmic *mper1* promoter activity, the amplitude of its oscillation is relatively low and therefore has not been optimal for distinguishing the properties of different rhythm-initiating treatments.

**Figure 1:**
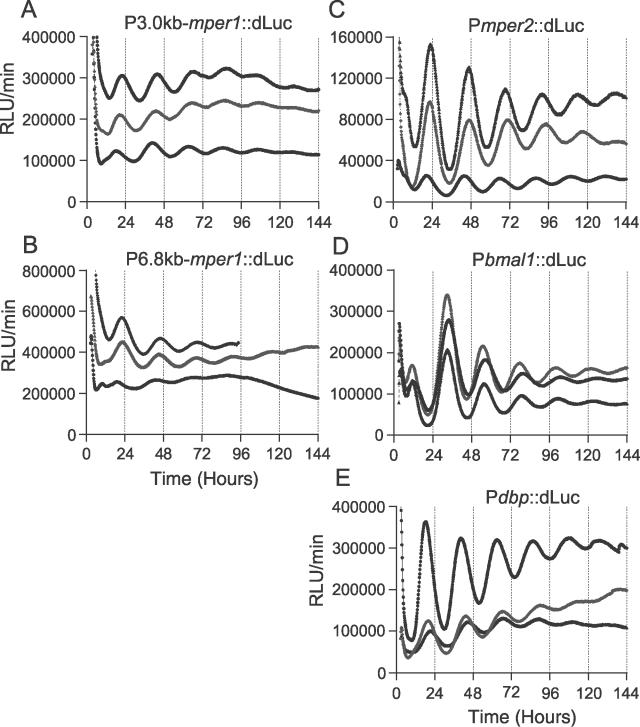
Bioluminescence Rhythms from Various Reporter Constructs That Were Stably Transfected into Rat-1 Fibroblasts Traces from three individual clones are shown for each reporter. Time 0 is the onset of 2-h Fsk treatment. All of the luminescence traces shown in this article, with the exception of Figure 4, are raw data that have not been manipulated. RLU/min: relative light units per minute. (A) Rat-1/P*mper1*::dLuc (3.0 kb *mper1* promoter). (B) Rat-1/P*mper1*::dLuc (6.8 kb *mper1* promoter). (C) Rat-1/P*mper2*::dLuc. (D) Rat-1/P*bmal1*::dLuc. (E) Rat-1/P*dbp*::dLuc.

To improve the reporter system, we modified both the promoter region and the luciferase region. For the promoter region, we first generated and tested a stable reporter line using a longer *mper1* regulatory region (6.8 kb) that contained five canonical E-boxes and was used to make a P*mper1*::Luc transgenic reporter rat [4,32] (Figure 1). Although the basal expression level increased with this reporter, there was no significant change in the oscillatory amplitude. Therefore, we tested the promoters of other genes: P*mper2* (1.7 kb from the transcription start site [33]), P*bmal1* (0.9-kb promoter region [−776 to +99][34]), and P*dbp* (5.0-kb regulatory region containing introns 1 to 3 [−579 to +4430 as a translational fusion] [35,36]). The temporal expression patterns of Rat-1 fibroblast cells that were stably transfected with these reporters all exhibited more robust circadian expression than those of the P*mper1* reporters (Figure 1). Unexpectedly, the *mper2* promoter, which has no canonical E-boxes but only a single noncanonical E-box at −23 relative to the transcription start site [37,38], showed high-amplitude rhythms. Of this second generation of reporters, we chose P*mper2*::dLuc for further studies in this paper, because (1) this reporter combination exhibited consistently robust oscillations, and (2) the mPER2 protein is one of the critical core clock components that has been implicated in resetting of the clock [39]. For this study, one reproducibly high expression line was selected (line no. 3–72, called “Rat1/P*mper2*::dLuc” hereafter).

**Figure 2:**
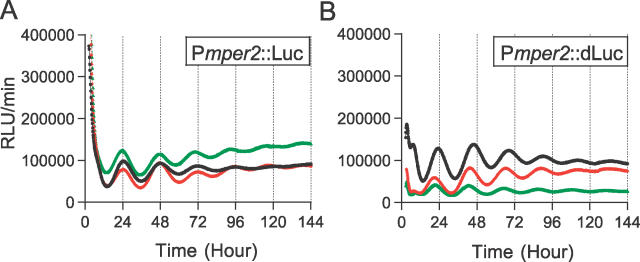
Comparison of Native Wild-Type Luciferase (Luc) versus Destabilized Luciferase (dLuc) Three individual clones are shown in each graph. Time 0 is the onset of 2-h Fsk treatment. (A) P*mper2*::Luc reporter. Note the high luciferase activity at the beginning of the monitoring. (B) P*mper2*::dLuc reporter. Note the absence of high luciferase activity at the beginning of the assay.

The original P*mper1*::Luc construct [15] used a native firefly luciferase reporter. When the half-life of this luciferase reporter was assessed by inhibiting protein synthesis with cycloheximide (50 μg/ml), it exhibited a half-life of 4.38 ± 0.16 h (*n* = 4) in the stably transfected Rat-1 cells. Because other groups have reported that the half-life of native luciferase in cultured SCN slices from P*mper1*::Luc transgenic mice is 1.2–1.4 h [40,41], we concluded that the relatively slower turnover of native luciferase in our system is characteristic of the Rat-1 fibroblast cells. To enable the measurement of more dynamic changes in circadian gene expression in vivo, we replaced the native reporter (“Luc”) with luciferase that has been destabilized by the fusion of a PEST sequence from a mouse ornithine decarboxylase gene to the C-terminal end of firefly luciferase cDNA (“dLuc”) [13,16]. Using Rat-1 cells that were stably transfected with a P*mper1*::dLuc construct, we measured the half-life of dLuc to be 2.83 ± 0.39 h (*n* = 5). Although the half life was shortened by only 1.5 h, the major benefit of using dLuc is that it shows no apparent accumulation of inactive luciferase in the absence of the luciferin substrate that causes the high basal activity of wild-type Luc during the first 12 h of recording (Figure 2; see also [42] for a description of this phenomenon in other systems). Therefore, dLuc is particularly useful for studies in which the first several hours of recordings are important (e.g., for monitoring signaling, synchronization, and rhythm initiation).

**Figure 3:**
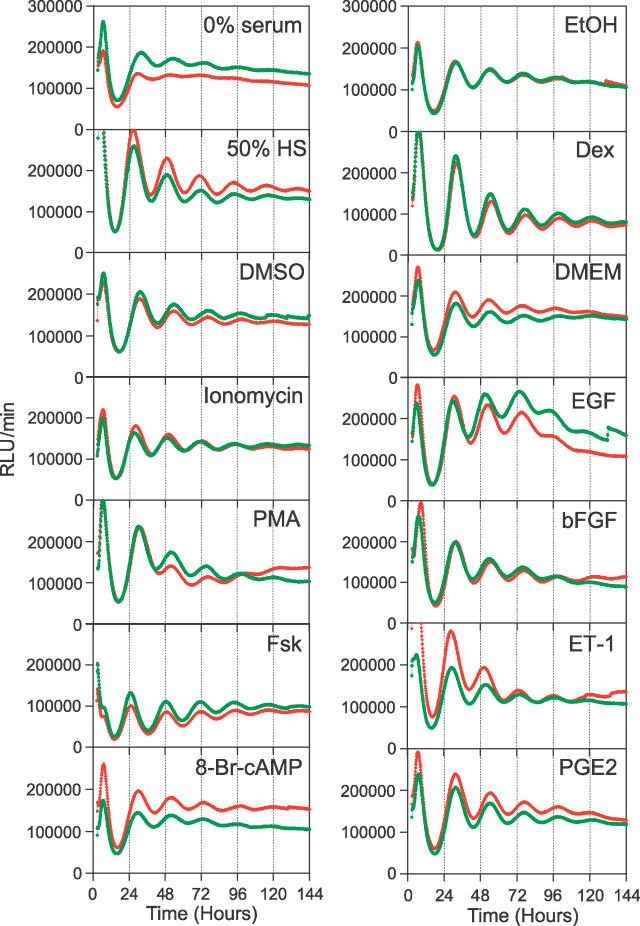
Fibroblast Rhythms Initiated by Various Treatments Confluent Rat1/P*mper2*::dLuc cells were treated for 2 h, then luminescence monitoring was begun. Time 0 is the onset of each treatment. DMSO (0.1%) is the solvent control for 1 μM ionomycin, 1 μM PMA, and 10 μM Fsk. Serum (0%) is a control for 50% horse serum. EtOH (0.001%) is the solvent control for 100 nM Dex. DMEM is the solvent control for 100 μM 8-bromo-cAMP, 30 nM EGF, 25 ng/ml bFGF, 30 nM ET-1, and 1 μM PGE_2_.. Two representative traces from one experiment are shown in each graph.

### Quantitative Analysis of Damping Rhythms in Mammalian Cells

Many different stimuli are capable of initiating rhythmicity in cultured cells. These include serum [7], Dex [43], Fsk [44], PMA, fibroblast growth factor (FGF), and EGF [8,10], calcium ionophores [10], endothelin-1 (ET-1) [45], glucose [12], and prostaglandin E_2_ (PGE_2_) [17]. The characteristics of the rhythms induced by these different treatments have been reported from a variety of studies from different labs (using different techniques and cell lines), and it is therefore impossible to compare the relative efficacy of these treatments for initiating circadian rhythmicity. In Rat-1 fibroblasts, serum, Dex, Fsk/cAMP responsive element binding protein (CREB), PMA, FGF, EGF, calcium, and glucose signaling pathways are known to be operating [7,10,12,43,44]. The ET-1 response pathway is also present in Rat-1 cells (unpublished data). NIH3T3 cells respond to PGE_2_ [17], implying that this pathway exists in NIH3T3 cells, but it is not known if this pathway is operative in Rat-1 fibroblasts. To quantitatively characterize the signaling pathways involved in the initiation of rhythmicity of Rat-1 fibroblast cells, Rat1/P*mper2*::dLuc cells were exposed to ten previously reported treatments for 2 h and assayed for the appearance of circadian rhythms in the real-time reporting system. As shown in Figure 3, we observed very significant variation in the subsequent rhythmic expression patterns induced by the different treatments.

The principal advantages of the real-time monitoring of luminescent cell cultures are the ability to obtain data that are highly resolved in time and to measure multiple samples in high-throughput mode. However, quantitative analyses of circadian rhythmicity in the Rat-1 fibroblast system are technically challenging because of the strong damping trend in the oscillations (i.e., variance non-stationarities). In addition to damping, these Rat-1 rhythms also exhibit a strong tendency for baselines to drift (i.e., mean non-stationarities). Furthermore, as shown by Figure 3 as well as by our previous report [15], different treatments evoke variable circadian gene expression patterns, and even the solvent controls or medium changes induce a certain degree of rhythmicity that is difficult to differentiate from some of the experimental treatments.

**Figure 4:**
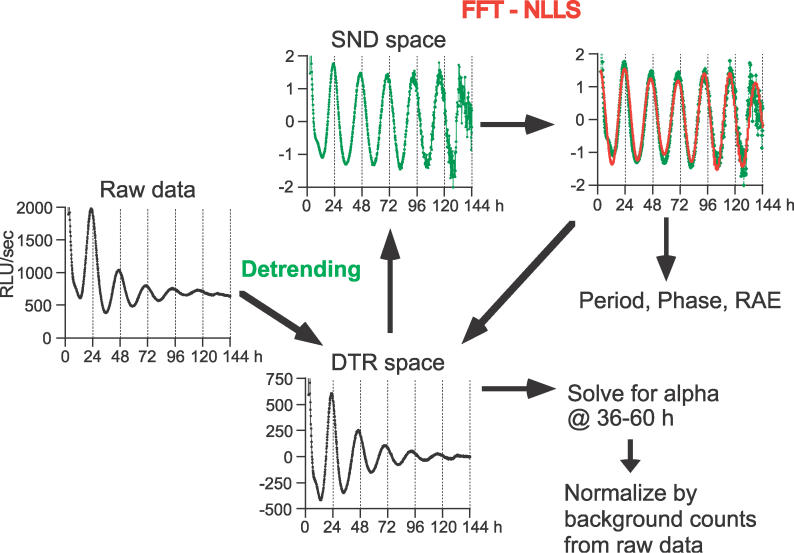
Schematic Representation of the Analysis Procedure One of the traces depicted in Figure 7 is shown. Raw data were first detrended by subtracting a 24-h moving average with uniform weight from raw data to produce zero-mean, zero-slope data. These subtracted data (DTR space) are further divided by the standard deviation to report the normalized detrended data in the SND space. These SND data then had a variance of 1, and were analyzed by FFT-NLLS to obtain period, phase, and RAE values. The figure shows an example of the fitted curve (green line, detrended raw data; red line, FFT-NLLS fitted curve). Based on these period and phase estimates, FFT-NLLS is performed on the subtracted data in the DTR space to solve for amplitude (α) between the fixed time range (36–60 h was used).

To analyze these time-series data, we employed step-wise quantification methods as follows: (1) detrending the luminescence time series data to extract rhythmic components; (2) FFT-NLLS analysis coupled with an RAE assessment to quantitatively determine rhythmicity; (3) estimation of period and phase by FFT-NLLS; and (4) a comparative analysis of oscillatory strength by deriving a measure of absolute amplitude and of normalized amplitude. FFT-NLLS is a multicomponent cosine fit analysis designed to objectively extract periods and phases from relatively noisy data sets at a user-specified confidence level (usually 95%) [25]. Most importantly, FFT-NLLS is coupled to a statistical assessment of the rhythmicity by a measure of RAE [25]. In addition to the period, phase, and RAE, we were also interested in calculating the magnitude of the observed luminescence oscillations, as we found that there was a distinct trend in the amplitude of circadian expression induced by various treatments (Figure 3). Differentiating gene expression patterns on the basis of magnitudes of oscillatory amplitude allows discrimination of weak versus strong synchronizing treatments. We therefore developed a method to assess the absolute amplitude and the normalized amplitude as described in Materials and Methods and Figure 4. This amplitude estimation successfully differentiated the effects of various treatments (Table 1 and see below).

**Table 1:**
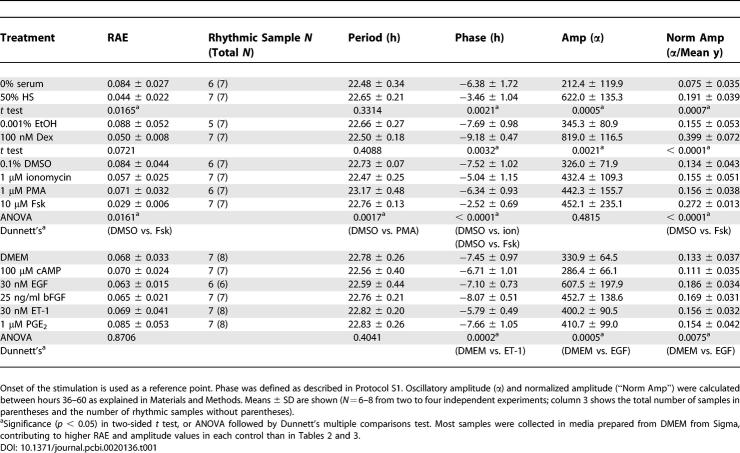
Summary of Quantification Analysis of Experiments in Figure 3

**Figure 5:**
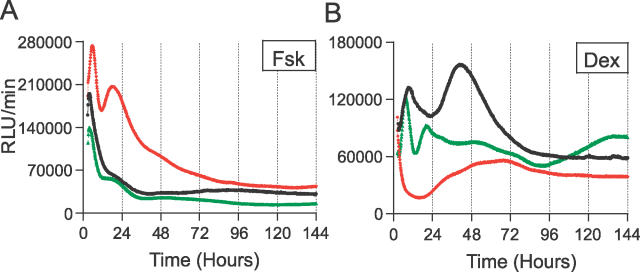
Representative Traces of the Rat-1 Cells Stably Transfected with P*sv40*::dLuc Time 0 is the onset of each treatment. Traces from three separate transfections are shown. (A) Rat-1/P*sv40*::dLuc cells treated with 10 μM Fsk for 2 h. (B) Rat-1/P*sv40*::dLuc cells treated with 100 nM Dex for 2 h.

As shown in Figure 3, some “control” treatments exhibited low-amplitude rhythms. In order to statistically judge whether these rhythms are significant or not, we used a reporter construct that is known a priori to be arhythmic to define an RAE threshold (as in [46]). As shown in Figure 5, by processing the time-series data from stable Rat-1 lines expressing luciferase under the control of the promoter from the SV40 gene (Rat1/P*sv40*::dLuc) which has been reported to be constitutively active [13], the critical RAE was determined to be 0.123 (see Materials and Methods for details). The traces shown in Figure 5 are representative of the large variability exhibited by the P*sv40*::dLuc reporter. From this point forward, Rat-1 samples whose RAE value was <0.123 were accepted as rhythmic at the 95% confidence level.

### Quantitative Analysis of Rhythms Induced by Various Treatments, Especially Fsk and Dex

**Figure 6:**
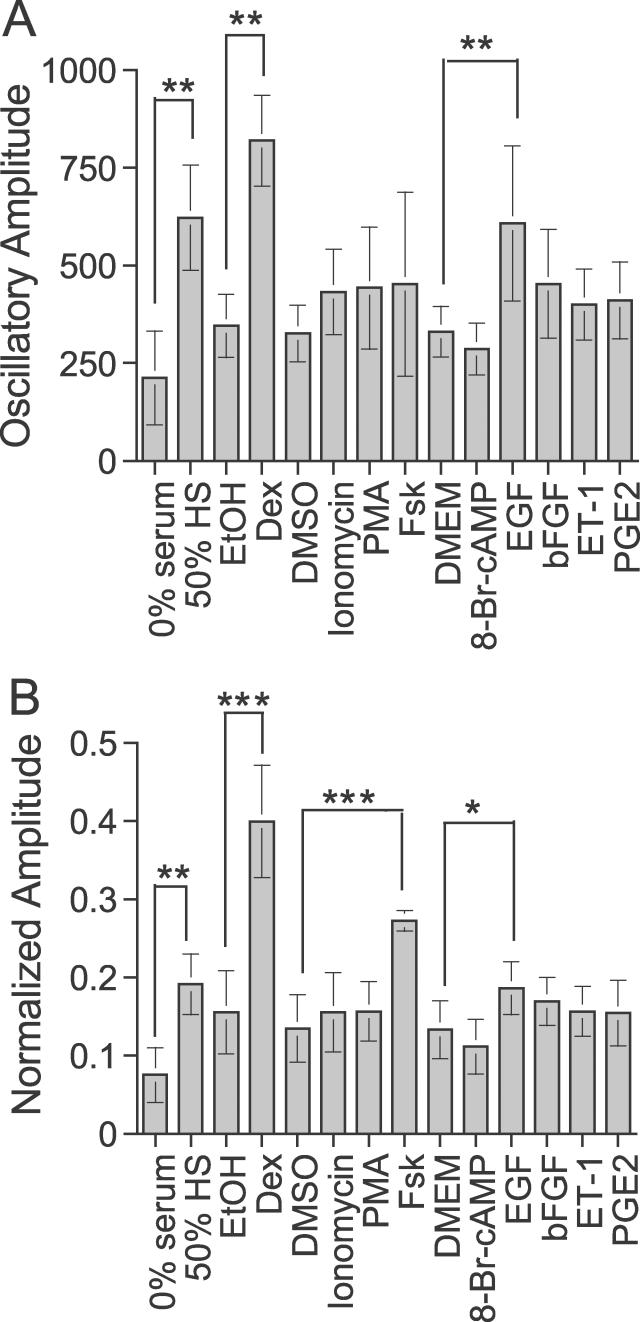
Histogram Representation of the Oscillatory Amplitude Calculations in Table 1 Error bars are ± S.D. *Statistical significance (*p* < 0.05) as compared to each control. **p < 0.005; ***p < 0.0005. (A) Absolute oscillatory amplitude. (B) Normalized oscillatory amplitude.

Using the analytical procedures described above, we quantified the rhythms induced by various treatments, and examined the relationship between signaling pathways and initiation of rhythmicity in Rat1/P*mper2*::dLuc cells (Figure 3 and Table 1). All the treatments induced a significant level of rhythmicity (RAE < 0.123). Among the ten compounds tested, horse serum, Dex, Fsk, and EGF were the most effective in generating rhythms with large oscillatory amplitude (Figure 6); Fsk induced a relatively higher degree of rhythmicity (RAE = 0.029; Table 1) than other treatments. In Figure 6, “normalized amplitude” gives the best indication of the efficacy of Fsk, possibly because the overall luminescence level of Fsk-treated cultures tends to be low and therefore the unnormalized amplitude is depressed.

Among these four efficacious treatments, we focused on Fsk and Dex for further analysis, as they induced the highest amplitude rhythms in Rat-1 fibroblasts (Table 1 and Figure 6B). In addition, the actions of both Fsk and Dex have been well characterized. Fsk is an activator of adenylyl cyclase in the cAMP signaling pathway and a potent inducer of CREB phosphorylation, and Dex is an analog of the endogenous ligand (glucocorticoid) for the glucocorticoid receptor (GR), a transcription factor that belongs to a nuclear receptor superfamily.

**Figure 7:**
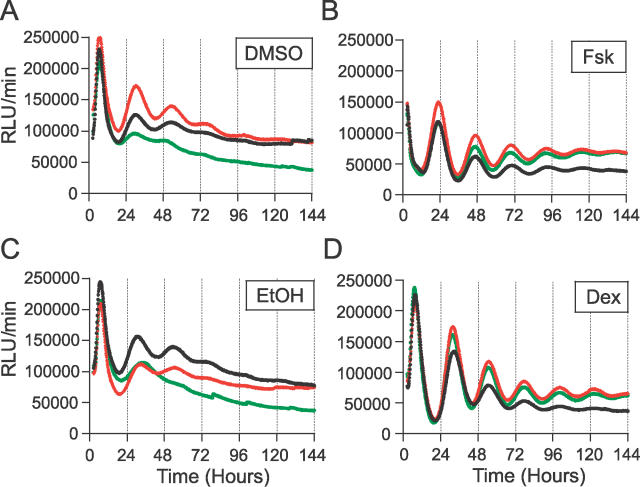
Stimulation of High-Amplitude Rhythmicity in Rat-1 Cells Rat-1/P*mper2*::dLuc reporter cells were treated with either solvent controls (DMSO or EtOH) or the specified treatments (Fsk or Dex) for 2 h before beginning to monitor luminescence. Time 0 is the onset of stimulation. Three replicates for the same treatments are shown in each graph. (A) 0.1 % DMSO (solvent control for 10 μM Fsk). (B) 10 μM Fsk. (C) 0.001% EtOH (solvent control for 100 nM Dex). (D) 100 nM Dex.

Solvent controls (0.1% DMSO and 0.001% EtOH) induced expression patterns that were possibly rhythmic (Figure 7A and 7C). According to the FFT-NLLS analysis, however, the rhythmicity in these DMSO/EtOH samples was barely significant (average RAE = 0.142 and 0.118, respectively). Individual DMSO/EtOH samples that exceeded the “rhythmicity threshold” showed only a minimal oscillatory amplitude (Table 2). These RAE results are in close agreement and, hence, consistent with our empirical determination of 0.123 as an effective RAE threshold for rhythmic determination. On the other hand, when stimulated by 10 μM Fsk and 100 nM Dex for 2 h, Rat1/P*mper2*::dLuc cells exhibited a high degree of rhythmicity (average RAE = 0.050 and 0.051, respectively) as well as high-amplitude oscillations that were significantly stronger than either control sample (Figure 7 and Table 2). Close inspection of period, phase, and amplitude between Fsk-treatment and Dex-treatment revealed two characteristics: (1) both the absolute amplitude and the normalized amplitude were consistently larger for Dex-initiated rhythms than for Fsk-initiated rhythms (Table 2), and (2) there was a significant phase angle difference elicited by the two treatments—the phase of the Dex-initiated rhythms was delayed by 8.37 h relative to that of the Fsk-initiated rhythms (*p* < 0.0001).

**Figure 8:**
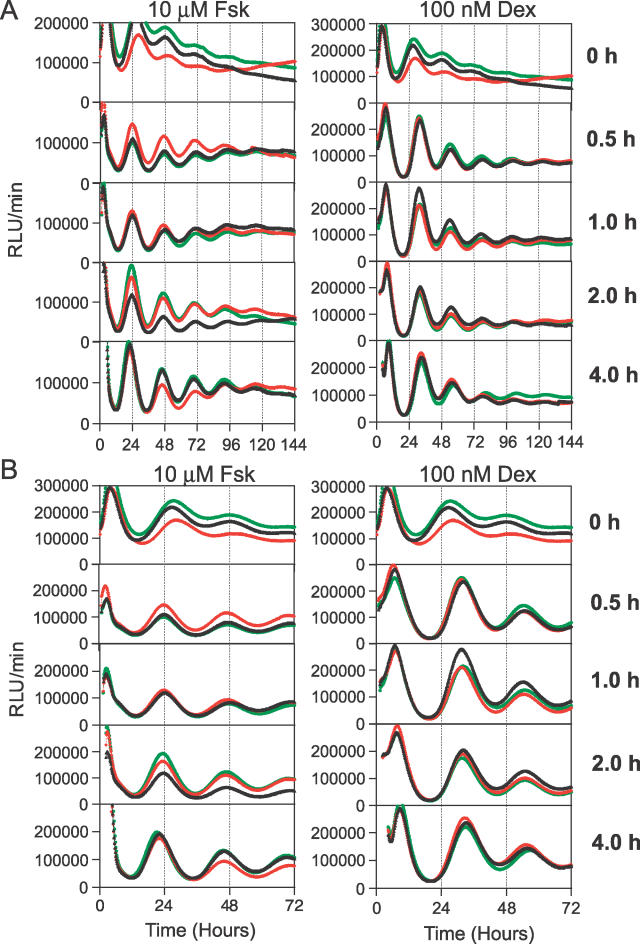
Time-Dependent Stimulation Effect of Two Drugs on the Initiation of Rhythmicity in Rat-1/P*mper2*::dLuc Reporter Cells Rat-1/P*mper2*::dLuc reporter cells were treated with either 10 μM Fsk or 100 nM Dex for the indicated period of time before monitoring luminescence rhythms. 0 h indicates no drug treatment (only a medium change). Time 0 is the onset of stimulation. Three replicates from a larger data set of the same treatments are shown in each graph (total *N* = 6). (A) Bioluminescence traces of 6 d of continuous measurements. Left panel, 10 μM Fsk; right panel, 100 nM Dex. The graph for the “0 h” control data is repeated with different ordinal scales for the Fsk and Dex columns to aid visualization (the ordinal scale is expanded for the Fsk column). (B) Figure 6A is rescaled to show the first 3 d. The scale for the ordinate is held constant in this panel.

**Figure 9:**
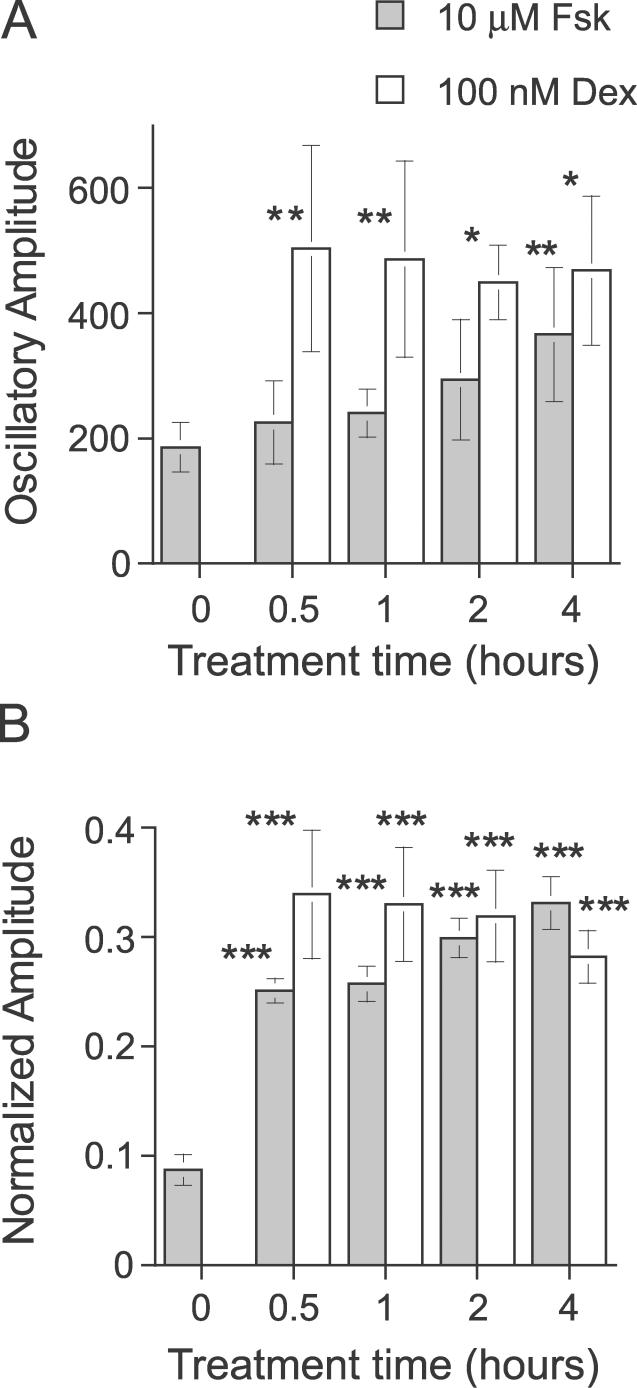
Histogram Representation of the Oscillatory Amplitude Calculations in Table 2 The gray bar is from 10 μM Fsk data; the clear bar is from 100 nM Dex data. Error bars are ± SD. *Statistical significance (*p* < 0.05) as compared to the medium change (0 h) control. (A) Absolute oscillatory amplitude. (B) Normalized oscillatory amplitude.

To further analyze the Fsk- and Dex-stimulated rhythms, we extended the assay to a detailed time-course analysis. A simple medium change (as indicated by treatment time = 0 h) initiated weak oscillations, but the degree of rhythmicity and amplitude of those rhythms were substantially less significant than those of Fsk- or Dex-stimulated cells, albeit just below our empirical RAE cutoff value of 0.123 (Table 3; average RAE = 0.105). Treatments with either Fsk or Dex (even for only 30 min) initiated significant oscillations (Figure 8 and Table 3). There was a significant difference between the periods of Fsk- vs. Dex-stimulated rhythms for 1-h treatments (Table 3, and also in Table 1), but these period differences were not significant for 0.5-, 2-, or 4-h treatments (nor were the periods of Fsk- vs. Dex-stimulated rhythms significantly different in Table 2). In addition to the observations noted for Figure 7, longer-duration treatments of Fsk elicited higher-amplitude oscillations (Figure 8 and Figure 9, ANOVA *p* = 0.030 among Fsk-treated samples). On the other hand, Dex treatments provoked the strongest oscillatory amplitude for 30-min treatments, and the oscillatory amplitude did not significantly increase with longer treatments, suggesting that 30-min treatments were already saturating (Figure 8 and Figure 9, ANOVA *p* = 0.91 among Dex-treated samples). Moreover, the oscillatory amplitude of Dex-induced rhythms was consistently larger than with Fsk-induced rhythms (Figure 8 and Figure 9). In contrast to Fsk treatments, which downregulated *mper2* promoter activity soon after washout, *mper2* promoter activity continued to climb for several hours after the termination of Dex treatments (Figure 7 and Figure 8). This phenomenon may relate to the observation that the phase of Dex-induced rhythms was consistently delayed compared to that of Fsk-induced rhythms (7.44 h later in the case of 2-h treatments with Fsk vs. Dex; Figure 8 and Table 3). When cells were treated with both Fsk and Dex, the final phase was intermediate between that by Fsk or Dex (4.7 h later than the Fsk-only phase and 2.3 h earlier than the Dex-only phase; see below). These results clearly suggest that the mechanism underlying synchronization by Fsk is different from that of Dex.

### Kinetics of Acute Promoter Responses Stimulated by Fsk and Dex

**Figure 10:**
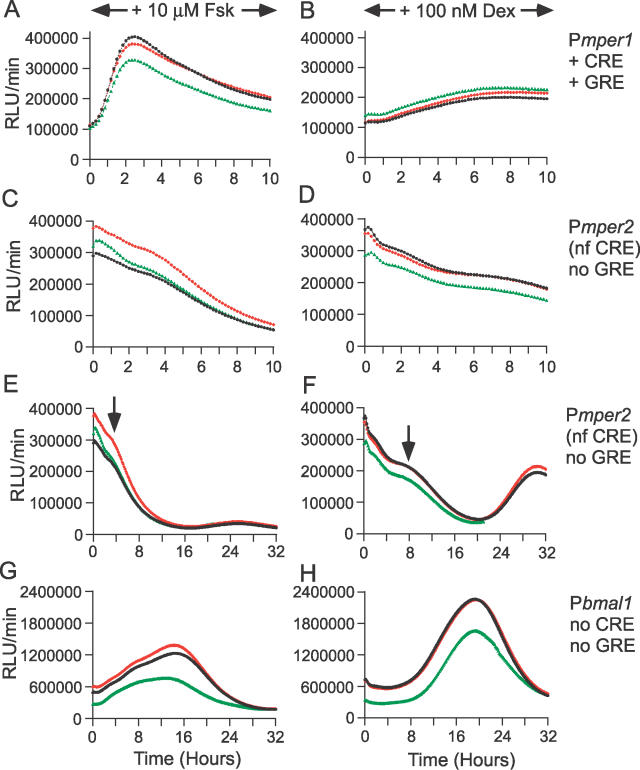
Effect of Continuous Stimulation by Fsk and Dex Promoter activities of P3.0kb-*mper1*, P*mper2*, P*bmal1* (Rat-1 cell line nos. 5–100, 3–72, and 5–66, respectively) were monitored in the continuous presence of either 10 μM Fsk or 100 nM Dex. Time 0 is when the Fsk/Dex was added. Three replicates are shown for each treatment. (A) Rat-1/P3.0kb-*mper1*::dLuc cells in the continuous presence of 10 μM Fsk. (B) Rat-1/P3.0kb-*mper1*::dLuc cells in the continuous presence of 100 nM Dex. (C) Rat-1/P*mper2*::dLuc cells in the continuous presence of 10 μM Fsk. NF, nonfunctional. (D) Rat-1/P*mper2*::dLuc cells in the continuous presence of 100 nM Dex. (E) Same as (C), except the time scale was expanded to 32 h. Arrows indicate the initiation of rhythmic components. (F) Same as (D), except the time scale was expanded to 32 h. Arrows indicate the initiation of rhythmic components. (G) Rat-1/P*bmal1*::dLuc cells in the continuous presence of 10 μM Fsk. (H) Rat-1/P*bmal1*::dLuc cells in the continuous presence of 100 nM Dex.

The *per1* gene has been implicated in phase-resetting, and indeed, *per1* transcription can be induced by a variety of signals [10,33]. The *per1* gene might also be a useful gauge of signal transduction triggered by Fsk and Dex, because the *mper1* promoter contains functional CRE and glucocorticoid response element sites within the 3.0-kb region [33,47]. We examined the acute effect of Fsk and Dex on *mper1* promoter activity in Rat1/P3.0kb-*mper1*::dLuc cells (Figure 10A and 10B). Continuous treatment with Fsk rapidly and strongly activated the *mper1* promoter, and the maximum activity level (3.4-fold, [3.40 ± 0.24, *n* = 3]) was reached 2.4 h (2.44 ± 0.10, *n* = 3) after Fsk was added. On the other hand, Dex took 8.5 h (8.50 ± 0.44, *n* = 3) to increase the *mper1* promoter activity to a peak value, which was only 1.8-fold (1.82 ± 0.11, *n* = 3) larger than the initial level. Unlike the rapid inactivation exhibited by Fsk treatment, Dex treatment led to a prolonged activation (see the plateau after 8.5 h).

We also examined the effects of continuous treatments with Fsk and Dex on the activity of *mper2* and *bmal1* promoter/reporters. The 1.7-kb region of the *mper2* promoter contained a nonfunctional CRE site [33] and no canonical glucocorticoid response element site. Moreover, we found neither CRE nor glucocorticoid response element consensus sequences on the 0.9-kb promoter of *bmal1*. As expected, neither promoter was affected acutely by Fsk and Dex (Figure 10C–10H; note that the time scales of Figure 10A–10D are 0–10 h, whereas the time scales of Figure 10E–10H are 0–32 h.) The effects of Fsk and Dex on the long-term *circadian* expression of the *mper2* and *bmal1* promoter/reporters were similar to that described before: earlier phase and low-amplitude oscillations initiated by Fsk, and delayed phase and high-amplitude oscillations stimulated by Dex (Figure 10C–10H).

### PRCs for Fsk and Dex treatments

**Figure 11:**
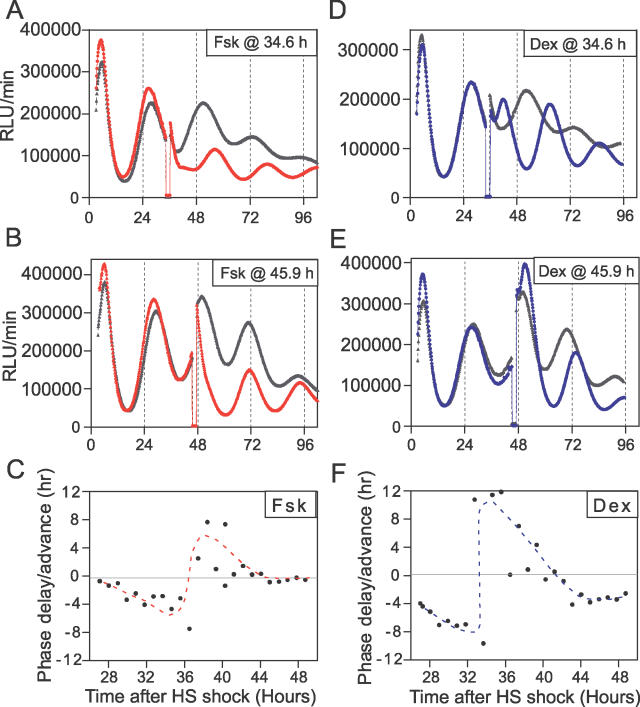
PRCs for Fsk versus Dex in Rat-1 Cells Confluent Rat-1/P*mper2*::dLuc cells were shocked with 50% horse serum at time 0. At the indicated circadian times during the second cycle (Day 2), cultures were treated for 2 h with either 10 μM Fsk (red line) in parallel with its solvent control (0.1% DMSO, gray line) or with 100 nM Dex (blue line) in parallel with its solvent control (0.001% EtOH, gray line). A representative trace for treatment and control is shown in panels (A), (B), (D), and (E). PRCs are plotted as phase shifts on the ordinate (delay shifts plotted as negative values, advance shifts as positive values) versus time after the horse serum (HS) treatment on the abscissa. (A) Pulse (2 h) of 10 μM Fsk or of 0.1% DMSO control at hour 34.6. (B) Pulse (2 h) of 10 μM Fsk or of 0.1% DMSO control at hour 45.9. (C) PRC for 10 μM Fsk pulses. Phases were corrected for the shifts by 0.1% DMSO. (D) Pulse (2 h) of 100 nM Dex or of 0.001% EtOH control at hour 34.6. (E) Pulse (2 h) of 100 nM Dex or of 0.001% EtOH control at hour 45.9. (F) PRC for 100 nM Dex pulses. Phases were corrected for the shifts by 0.001% EtOH.

**Figure 12:**
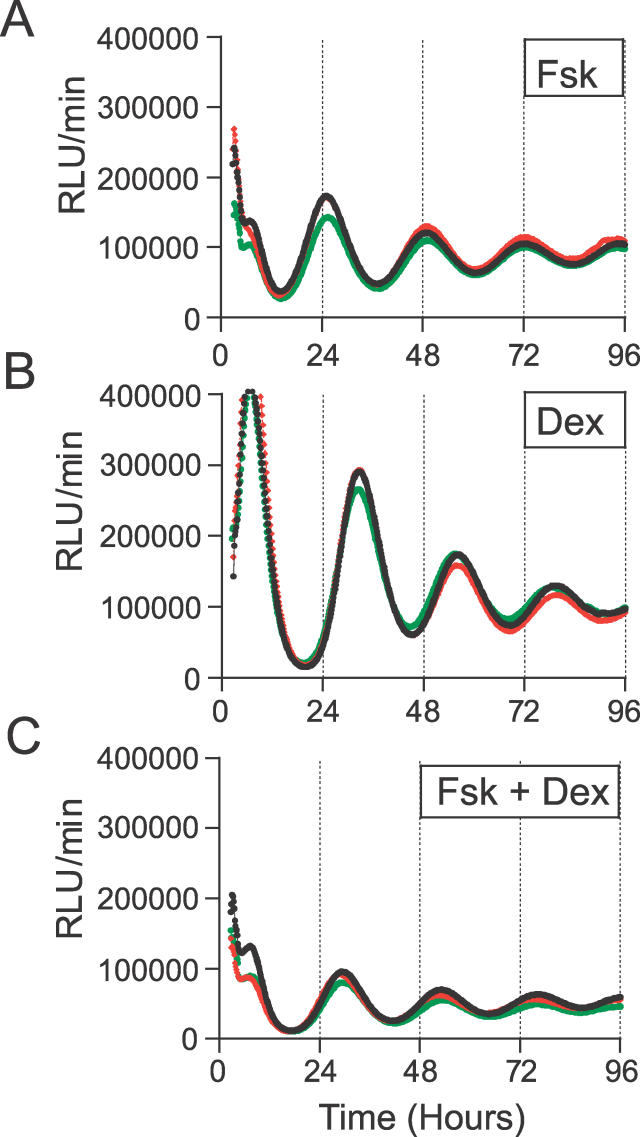
Comparison of Phases Initiated by a Combination of Fsk and Dex Stimuli Rat-1/P*mper2*::dLuc cells were treated with 10 μM Fsk or 100 nM Dex or 10 μM Fsk + 100 nM Dex for 2 h. The phases of rhythms initated by each treatment were: Fsk = −2.92 ± 0.79 (*n* = 5), Dex = −9.95 ± 0.26 (*n* = 6), and Fsk + Dex = −7.60 ± 1.26 (*n* = 5). Time 0 is the onset of stimulation. Three replicate measurements are shown for each treatment. (A) 10 μM Fsk. (B) 100 nM Dex. (C) 10 μM Fsk + 100 nM Dex.

In addition to the kinetics of action, differences in the phase of oscillations initiated by Fsk vs. Dex could be related to their phase-resetting properties. This possibility was tested by measuring phase response curves (PRCs) to pulses of Fsk or Dex. Confluent cultures of Rat1/P*mper2*::dLuc cells were shocked with 50% horse serum, and treated with 2-h pulses of 10 μM Fsk and 100 nM Dex (in parallel with pulses of solvents as controls) at different phases during the second cycle (day 2). The phases corrected for the minor shifts evoked by the solvent controls were plotted in hours after the serum shock and depicted in Figure 11. Comparison of the Fsk-stimulated PRC with that stimulated by Dex showed that the magnitude of the phase-shifts caused by 10 μM Fsk is smaller than those by 100 nM Dex and that the two PRCs are phased differently—the “breakpoint” for the Fsk PRC is at approximately 37 h after serum shock, whereas that for Dex is about 3 h earlier, at approximately 34 h after serum shock (Figure 11). Because the PRCs for Fsk vs. Dex are phased differently, the phase of Rat-1 cells treated with these stimuli will initiate rhythms with different phases, as observed in Figures 3, 7, and 8 and as tabulated in Tables 1–3. What happens when cells are treated with both Fsk *and* Dex? Figure 12 shows that costimulation with both Fsk and Dex results in a phase relationship that is intermediate between those evoked by Fsk or Dex alone: the phase elicited by Fsk is −2.92 ± 0.79 (*n* = 5), and that elicited by Dex alone is −9.95 ± 0.26 (*n* = 6), whereas the rhythms initiated by Rat-1 cells costimulated by both Fsk and Dex establish an intermediate phase of −7.60 ± 1.26 (*n* = 5). A *t* test showed a significant difference (*p* < 0.01) between Fsk vs. Fsk + Dex and between Dex vs. Fsk + Dex.

## Discussion

### Different Treatments Evoke Rhythms with Varying Characteristics

The present study demonstrates that the various treatments used by previous researchers to initiate rhythms in fibroblasts can lead to a variety of oscillatory amplitudes and phases (Figure 3). As assessed by RAE values (considering only those with RAE < 0.123, indicative of statistically significant rhythmicity), rhythmicity was initiated by all ten compounds presently considered (serum, Dex, ionomycin, PMA, Fsk, 8-bromo-cAMP, EGF, basic FGF [bFGF], ET-1, and PGE_2_). Whereas the solvent controls also elicited weak oscillations, in all likelihood these rhythms arose not as a consequence of the solvents per se, but rather the transfer from growth medium (containing 5% fetal bovine serum [FBS]) to fresh assay medium (containing 10% FBS), a procedure employed for both control and experimental samples (see also [12]). These weak oscillations, induced by media transfer, were characterized by RAE values very similar to the RAE values determined empirically to be expected for constitutively expressed markers (i.e., SV40) in this Rat-1 fibroblast model system.

Our amplitude analyses indicated that serum, Dex, Fsk, and EGF were the most effective inducers in generating high-amplitude rhythms in Rat-1 cells. Intriguingly, these treatments also initiated rhythms with different phase relationships (see below). Based upon a predicted ultimate action on CREB phosphorylation and transcription activation from CRE sites [33], we initially expected that Fsk, 8-bromo-cAMP (a potent protein kinase A activator), EGF, bFGF (factors for the receptor tyrosine kinase–mediated mitogen-activated protein kinase pathway), and ET-1 (an activator of the protein kinase C–activated mitogen-activated protein kinase pathway) would have had relatively equivalent effects. However, of this group of activators, only Fsk and EGF initiated statistically significant rhythms (of significantly higher amplitude than the nonsignificant rhythms elicited by 8-bromo-cAMP, EGF, bFGF, and ET-1) (Figures 3 and 6, and Table 1).

These differences are likely to be due to both (1) different efficacies in activating the CREB phosphorylation pathway and (2) the certitude that many of these treatments activated various secondary pathways in addition to those that terminate on CREB phosphorylation. For example, direct addition of the substrate cAMP (8-bromo-cAMP) usually requires relatively high concentrations (in the range of 0.1–2.0 mM) to elicit a response so as to overcome poor membrane permeability. Fsk, on the other hand, at much lower concentrations (1–20 μM), enhances the activity of adenylyl cyclase, the enzyme that subsequently produces more locally high concentrations of cAMP that can act upon protein kinase A.

**Table 2:**
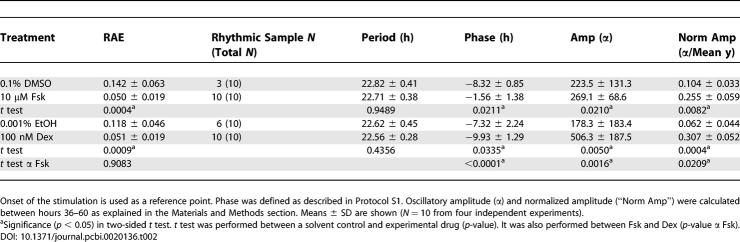
Summary of Quantification Analysis of Experiments in Figure 7

**Table 3:**
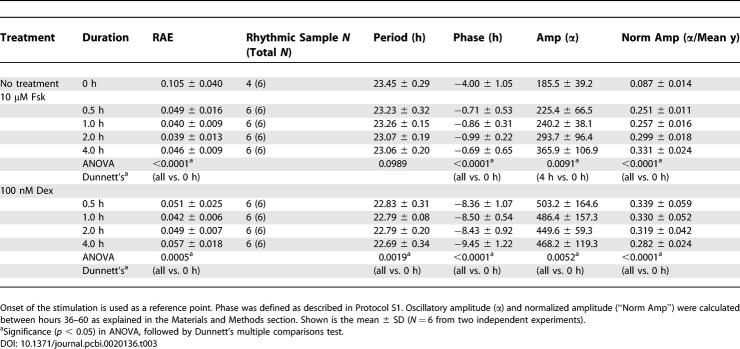
Summary of Quantification Analysis of Experiments in Figure 8

Another reason for the observed differences in efficacy among the CREB-activating treatments and for the differences between our study and other studies is probably related to the fact that the cell line used in our investigation (Rat-1 cells) likely expresses a different ensemble of genes encoding either receptors and/or downstream signaling-pathway proteins than did the NIH3T3 cells used by Akashi and Nishida [8] or the primary fibroblasts used by Yagita et al. [45]. Specifically, Rat-1 cells do not express the PGE_2_ receptor EP-1, whereas NIH3T3 cells do [17,23]. Therefore, NIH3T3 cells have the potential to respond to PGE_2_ robustly, whereas Rat-1 cells would not be expected to do so (Table 1). Finally, another explanation for the differences between our study and other studies may be related to differences in the ingredients used in the assay medium. For example, in our previous paper [15], we compared the inclusion of FBS with the serum-free supplement B27. When B27 is included in the assay medium, simple medium changes elicit significant rhythmicity [15,48]. Some earlier studies used serum-free medium, and it is possible that our inclusion of 10% FBS in this investigation may have masked relatively subtle effects that were elicited by treatments used in prior studies using serum-free medium. We included FBS in our assay medium because it allows cell viability to be extended so that we can measure more cycles. Finally, the source of DMEM can affect the assay; we have found a difference in the free-running rhythm when Rat-1 cells are assayed in DMEM obtained from GIBCO vs. Sigma (see Table 1 and Materials and Methods), and there might be other unreported consequences of using DMEM from different sources.

### Comparison of Quantitative Methods for Analyzing Rhythmic Patterns

Our present conclusions were made possible by a quantitative analysis that employed a combined approach in which the FFT-NLLS rhythms analysis procedure [25] was conducted along with an objective data preprocessing strategy that successfully eliminated mean and variance non-stationarities in our observed P*mper2*::dLuc reporter rhythms. The implementation of this combined strategy permitted reliable assessment, in objective statistical terms, of both (1) the degree of expressed rhythmicity (as assessed by a statistical measure of rhythmic determination, the FFT-NLLS RAE) and (2) the absolute and normalized magnitude of oscillatory amplitude. Both goals were achieved because this strategy not only (1) objectively eliminated baseline drift (i.e., mean non-stationarities), but also (2) surmounted the otherwise confounding complications of time-dependent oscillatory damping (i.e., variance non-stationarities), both of which have been reported previously as common occurrences in observed peripheral tissue rhythms [4,15]. As a consequence, we were able to successfully assess, on a statistical basis, the strength of the various treatments in both (1) initiating ensemble rhythmicity as well as (2) the magnitude of the resulting oscillations.

There are two major differences between the current method described herein and the previous FFT-NLLS method for analyzing chronobiological data that was created by one of us (M.S.) [25]. One difference is the application of an aggressive detrending procedure that effectively reduces mean and variance non-stationarities in a model-independent manner prior to the FFT-NLLS analysis (this part of the procedure is called DTRNDANL—see Protocol S1). We found this to be necessary because the extent of the non-stationarities in our original data was such that analysis without the DTRNDANL preprocessing produced results prone to bias (i.e., FFT-NLLS was unable to handle the original data reliably). The other difference from the previous method [25] is that the current procedure also characterized absolute oscillatory amplitude magnitudes for subsequent interpretation. This was done by implementing the concept underlying FFT-NLLS, except restricted to first order (i.e., a single-cosine wave–functional representation).

Our data acquisition system in this study was the LumiCycle, marketed by Actimetrics (Wilmette, Illinois, United States). There is a convenient analysis package that is included with the LumiCycle for detrending and period/phase analyses. Our method described herein differs from that included with the LumiCycle in several respects. We use a more aggressive detrending algorithm (DTRNDANL) that is model independent and objective because the only user-specified variable is the filter period, whereas the detrending LumiCycle program models baseline drift as a polynomial of user-specified order (a subjective and potentially arbitrary process; see Protocol S1). Moreover, our analytical procedure performs a statistical assessment of model parameter confidence limits and rhythmic significance by way of RAE from the FFT-NLLS procedure (see Protocol S1). As described in Protocol S1, the LumiCycle's software includes several methods for period analysis, but most do not assess statistical significance or confidence (with the exception of chi-square periodograms, which can reliably produce assessments of significance, but only for truly mean- and variance-stationary data). The current analysis procedure has been optimized for (1) considering statistical issues regarding relatively subtle differences among conditions in chronobiological data and (2) discerning the magnitude of rhythmic amplitude or “strength” (in particular, RAE helps answer the question, “Is there a rhythm in that data…or not?”).

### What Does “Oscillatory Amplitude” Reflect?

The underlying basis for our observation that some treatments evoke higher amplitude rhythms than others is, in all likelihood, related to the contributions of two combined effects: (1) ensemble synchronization of cell populations; and (2) cellular factors influencing the rhythmicity of individual cells.

Regarding population effects, Schibler and coworkers [22] and Welsh and coworkers [48] recently reported bioluminescence imaging experiments on individual cells in which they persuasively demonstrated that rhythmic damping in their cell cultures was predominantly due to phase-desynchronization of individually oscillating cells. These reports indicated that the rhythmicity manifested in an ensemble of cultured cells is a result of the resynchronization of individually oscillating, but asynchronous, cells. By this mechanism, initiation of rhythmicity in cell cultures by signaling stimuli would be due to phase-resetting within a population of individually oscillating cells. As such, differences in oscillatory amplitude elicited by different treatments would be a reflection of the phase-shifting and coordinating efficacy of the various treatments.

The second relevant effect is cellular. Differences in oscillatory amplitude in cell cultures will also reflect the amplitude of the oscillation in each individual cell. In this case, the different stimuli that initiate circadian oscillations of different oscillatory strength would be inferred to invoke different genetic expression patterns that ultimately result in oscillations of “clockwork” genes of varying amplitude. On the basis of the change in waveform/bandwidth of P*mper1*::Luc rhythms as they damp in Rat-1 cell populations, we previously proposed that damping of the rhythms is possibly due to a progressive decrease of the Rat-1 pacemaker's amplitude in each cell over time [15]. Whereas the aforementioned studies suggest that desynchronization among oscillating cells is the predominant basis for the progressive decline of oscillatory amplitude in populations of cells [22,48], differential oscillatory amplitudes at the cellular level cannot be ruled out as also playing a contributory role.

### Two Distinct Pathways Can Initiate Circadian Expression in Fibroblasts

We found that both Fsk and Dex elicit high-amplitude rhythmicity in Rat-1 fibroblasts, so these cells must express receptor/signaling components that allow responsiveness to these two compounds (other studies confirm this conclusion [10,43]). However, quantitative analyses of Fsk- and Dex-induced rhythms revealed several distinct characteristics. A significant phase angle difference was found between Fsk and Dex treatments (7.44 h difference after 2-h treatments; Figure 8 and Table 3) that is attributable to the phase angle difference of PRCs evoked by these treatments (Figure 11). Also, the magnitude of the oscillatory amplitude was consistently larger with Dex treatment than with Fsk treatment for all incubation periods (Figure 8 and Table 3). Our time-course analyses of Fsk treatment demonstrated that longer incubation times elicited progressively larger oscillatory amplitudes (Figure 8 and Table 3), implying that 30-min treatment with 10 μM Fsk was below the saturation threshold and longer treatments are needed for effective population synchronization and/or cellular initiation by the cAMP pathway. On the other hand, Dex stimulation showed a different trend, whereby 100 nM Dex for 30 min appeared to be saturating for maximal population synchronization and/or cellular initiation. These phase and amplitude differences clearly suggest that the resetting and synchronization mechanisms of Fsk and Dex are different, as is graphically demonstrated by the PRCs shown in Figure 11.

These phase and amplitude differences are likely to be a consequence of (1) the *magnitude/kinetics* of the circadian gene expression that is activated, and/or (2) the different *repertoire* of genes activated by Fsk vs. Dex. These distinct patterns of gene expression subsequently orchestrate the resetting characteristics observed in the present study. *Per1* promoter activity serves as an indicator of the differential activation of core circadian clock genes, as well as of immediate early genes, both of which are important examples of coordinate regulation. Fsk is expected to act primarily via CREB phosphorylation, whereas Dex acts via the GR pathway, both of which ultimately modulate a wide variety of transcriptional activities [49–51]. Transcriptional activation by CREB and GR generally begins within 5–30 min of stimulus onset [44,50,52]. However, Fsk and Dex showed different kinetics in activating *per1*, in which Fsk showed rapid/strong activation whereas Dex showed slow/weak activation (Figure 10A and 10B). Our results are consistent with those of Balsalobre et al [10], who measured the induction of *rper1* mRNA in response to Fsk and Dex. They also found that Fsk rapidly induced *rper1* mRNA, reaching maximal levels at 1–1.5 h, while the Dex response was considerably slower [10]. For Fsk-induced rhythms, the rapid induction kinetics we observed (Figure 10) were likely due to acute induction of a set of CREB-induced genes (including *per1* itself), which subsequently rapidly initiate rhythmicity. On the other hand, the relatively slower kinetics of *per1* gene induction by the GR pathway are consistent with Dex-induced rhythms, manifesting a delayed phase. An additional component is that the level of genes that are activated by Fsk and Dex may affect circadian gene expression by negative feedback. For instance, *per1* is a repressor of circadian oscillations [1] and thus its strong induction (e.g., by 10 μM Fsk) may have a negative effect on the overall gene expression (e.g., [7,10,44]). Therefore, the differences between Fsk- and Dex-induced rhythms observed in this study are possibly the result of the treatment-specific kinetics of gene expression and of the different sets of genes that are activated by Fsk vs. Dex.

### Implications of Different Signaling Mechanisms Elicited by Fsk versus Dex

The phase angle difference we have observed between Fsk vs. Dex induction in Rat-1 cells may help explain the 3–9 h later phase of rhythms in peripheral tissues relative to those in the SCN [36,53,54]. Because the SCN lacks GRs [43,55,56], there is likely to be a fundamental difference in the resetting mechanisms in SCN relative to peripheral tissues (at least, with those expressing GRs). Photically stimulated CREB (via glutamate, PACAP, Ca^++^, and protein kinase A, etc.) likely plays a predominant role in SCN, whereas peripheral tissues are more likely to be responding to humoral signals that activate CREB pathways, as well as glucocorticoid pathways (and probably others as well). This concept is depicted in Figure 12. A tissue that is receptive to signaling by the CREB pathway alone might behave analogously to that of Rat-1 cells treated with Fsk alone, whereas a peripheral tissue that is responsive to CREB signaling and multiple other pathways might behave analogously to that of Rat-1 cells treated with both Fsk and Dex (Figure 12). The phase of cells treated with Fsk + Dex (and Dex alone) are later than those of cells treated with Fsk alone. These differential effects may help explain the specific phase angle differences among individual tissues and organs, each of which utilize unique signaling pathways based on different sets and distributions of expressed receptors and activators.

## Materials and Methods

### Plasmid constructs.

The promoter/regulatory region of each construct [15,32–34,36] along with firefly luciferase cDNA was subcloned into pcDNA3.1/Hygro^+^ vectors (Invitrogen, Carlsbad, California, United States) so that (1) the luciferase cDNA was in frame and (2) the 5′ end of the promoter was protected by transcription termination (see [15]). Destabilized luciferase (dLuc) was created by attaching a PEST sequence fragment from the mouse ornithine decarboxylase gene before the terminal codon of luciferase (as in [13,16]). For a constitutive reporter, pMOS/P*sv40*::dLuc was prepared so that the selection gene cassette (P*cmv*::hyg::SV40pA) preceded the reporter cassette (P*sv40*::dLuc::BGHpA), which was then capped by a transcriptional terminator. This design facilitated the identification of stable transformants and minimized possible effects of the integration site on the activity of the promoter of interest.

### Cell culture and stable transfection.

Rat-1 fibroblast cells (a generous gift from Dr. Michael Bishop) were cultured as previously described [15]. Stable transfection and selection of hygromycin-resistant colonies was also done as previously described [15], except that LipoFectamine 2000 (GIBCO, San Diego, California, United States) was used for transfection. To screen for stable P*mper2*::dLuc reporter lines, the following criteria were employed: (1) the level of the reporter's signal was required to be above the background of the recording equipment (> 100 cps in the LumiCycle, whose dark counts were 30–60 cps); (2) when assayed for circadian rhythmicity at 100% confluency, the stable clones were to exhibit a rhythm for at least two cycles, as confirmed by a transient transfection assay (unpublished data) and also as shown for *mper2* mRNA expression by Balsalobre et al. [7]; (3) there were to be no growth retardation or morphological alteration that may have arisen from the random insertion of the DNA construct; and (4) the clones were to exhibit a reproducible circadian expression assay. Most experiments reported here used a line called Rat1/P*mper2*::dLuc (line no. 3–72), which was one of the lines that reproducibly showed a high-amplitude luminescence rhythm.

### Real-time bioluminescence monitoring assay.

Fsk (F6886; Sigma, St. Louis, Missouri, United States) was dissolved in DMSO and Dex (D1756; Sigma) was dissolved in EtOH. Approximately 3–5 × 10^5^ cells/35-mm dish were seeded at least 6 d prior to the experiment. Before recording, the cells were treated with 10 μM Fsk (final DMSO concentration of 0.1%) or 100 nM Dex (final EtOH concentration of 0.001%) for the indicated period of time (2 h if not otherwise specified). At the end of each treatment, the medium was replaced with assay medium (DMEM lacking phenol red [catalog no. 13000–021; GIBCO] supplemented with 10% FBS [no. 16000–044; GIBCO], 10 mM HEPES [pH 7.2], antibiotics [50 U/ml penicillin, 50 μg/ml streptomycin], and 0.1 mM luciferin [Promega, Madison, Wisconsin, United States]). Because GIBCO stopped producing DMEM no. 13000–021 during this study, DMEM no. D2902 from Sigma supplemented with 3.5 g/l glucose and 350 mg/l NaHCO_3_ was used in later experiments (the change of source for the DMEM medium was responsible for some of the differences in the RAE values of Table 1 vs. Table 2 and Table 3; the data of Table 1 were obtained using DMEM from Sigma, while the data of Table 2 and Table 3 were obtained using DMEM from GIBCO). Dishes were sealed with 40-mm circular coverslips (40-mm circle no. 1, catalog no. 40CIR-1; VWR, West Chester, Pennsylvania, United States) and a bead of silicon grease.

All assays were done in a LumiCycle, which is a 32-channel automated luminometer that was placed within a 36.5 °C incubator in a temperature-controlled room. All samples were measured every 10 min with an integration time of 75 sec for at least 6 d.

### Quantitative analysis of bioluminescence data.

The raw data were first detrended to produce zero mean and constant (=1) variance. A sliding 24-h window was moved across the entire time course. A uniformly weighted linear regression correction (calculated within each of the successive sliding 24-h windows) was subtracted from each of the original raw data values and retained in program memory. This was done across each of the sliding windows affecting each point, after which each of the 24-h corrected values for each datapoint were averaged and subsequently stored with their corresponding *x* values. Data detrended by only this linear-regression correction strategy are referred to as residing in detrended only (DTR) space. To additionally correct for damping effects, each 24-h window of detrended data (after the linear-regression correction step) was further divided by the standard deviation within each corresponding sliding 24-h window and retained in program memory. Averaging was then performed again as described above to produce a detrended time series of constant unit variance (these data are referred to as residing in standard normal deviate [SND] space). This procedure removed mean non-stationarities (i.e., baseline drifting) from the raw data and, if the data were reported in SND space, variance non-stationarities (i.e., damping) were also removed (Figure 4).

After detrending, all data were analyzed for period and phase by FFT-NLLS analysis [25]. Onset of stimulation (e.g., Fsk, Dex, etc.), was used to define time zero. Data within the first 144 h after the onset of stimulation were considered in the analysis. Phase was defined as described in Protocol S1. In addition to period and phase estimation, the level of rhythmic determination was assessed by the RAE, which was obtained by dividing the amplitude error by the most probable amplitude estimate, and was expressed as a fractional value ranging from 0 to 1.0, where “0” corresponds to a rhythmic component known to infinite precision (zero error), and “1.0” corresponds to a rhythm that is not statistically significant.

Based on the period and phase information from the SND space analysis, oscillatory amplitude (α) was calculated by a linear least squares estimation of DTR space data, according to the following equation:

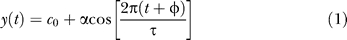
The measurement interval for our analyses in this paper was restricted to hours 36–60, which corresponds to the interval after the transient activation effect had decayed, but before significant damping of oscillatory amplitude was evident. The resultant absolute amplitude value was used to compare the absolute oscillatory strength exhibited by different samples. In order to compensate for a possible variation between samples (e.g., resulting from differences in total cell number, luminescence intensity, etc.), “normalized amplitude” was also computed by dividing the absolute amplitude (α) by background, which was calculated as an average of luminescence intensity between 36–60 h in the raw data.

An RAE threshold value was employed to distinguish between (1) the absence vs. (2) the presence of rhythmicity. The cut-off RAE value was determined essentially as in Stanewsky et al. [46]. In the mammalian case, we tested P*sv40,* a promoter that has been determined to be nonrhythmic [13]. Rat-1 cells were stably transfected with pMOS/P*sv40*::dLuc so that the linearized plasmids would be randomly integrated throughout the genome, and were subsequently pooled after selection. We prepared four independent transfections with three different concentrations of pMOS/P*sv40*::dLuc plasmid, and measured 96 samples for Fsk treatment and 88 samples for Dex treatments, as each drug treatment caused different expression patterning. Out of these samples, 95 and 88 samples were detected as rhythmic (periods ranged from 18–28 h). The average RAE of these samples was 0.275 ± 0.107 and 0.247 ± 0.105, respectively. Assuming a normal distribution, 5% of the lowest RAE values from Rat1/P*sv40*::dLuc represents the “strong rhythmicity level” of the SV40 promoter. Based on the assumption that the SV40 promoter is arhythmic, a critical RAE value was calculated to be 0.124 for Fsk treatment and 0.123 for Dex treatment. We used 0.123 to be the threshold to define rhythmicity at a 95% confidence level. In this study, data whose RAE value was <0.123 were judged to be rhythmic. For a more detailed description of our method of quantitative analysis as well as a brief description of the software analysis package included with the LumiCycle, please see Protocol S1.

## Supporting Information

Protocol S1 Detailed Descriptions of Data Analysis Methods Used by the Authors and as Included with the LumiCycle (36 KB DOC)

## Abbreviations

bFGF: basic fibroblast growth factor
CREB: cAMP responsive element binding protein
Dex: dexamethasone
DTR: detrended only
EGF: epidermal growth factor
ET-1: endothelin-1
FBS: fetal bovine serum
FFT-NLLS: fast Fourier transform–nonlinear least squares
Fsk: forskolin
GR: glucocorticoid receptor
PGE_2_: prostaglandin E_2_
PRC: phase response curve
RAE: relative amplitude error
SCN: suprachiasmatic nucleus
SND: standard normal deviate
