# Supplementary material for: Quantitative Analyses of Circadian Gene Expression in Mammalian Cell Cultures
Source: PLoS Comput Biol. 2006 Oct 13;2(10):e136. doi: 10.1371/journal.pcbi.0020136 (PMC1599765; doi:10.1371/journal.pcbi.0020136)
Supplement: Supplementary file 1 [file pcbi.0020136.sd001.doc]

**Supporting Information**

**Detailed Description of the Current Method for Data Analysis:**

As described in Material and Methods, our approach for data analysis consists of multiple steps: DTRNDANL, FFT-NLLS, and the derived RAE.

**DTRNDANL:**

Detrending (*i.e.*, removal of baseline drift) is accomplished by DTRNDANL in both a (i) model-independent and (ii) highly-objective manner in which the only user-specified variable is the filter period (chosen to be 24-hours in the current case of circadian data). DTRNDANL applies a detrending algorithm to time series data in which the user specifies three inputs:

(i) the value of a filter window (FWINDOW) to apply (24 h in the case of circadian data).

(ii) whether the detrended data are to be presented in original y-value-space or in terms of

standard normal deviates space (SND-space).

(iii) whether pointwise uncertainties are to be reported with the detrended data as

(a) all zeroes (*i.e.*, uniform weighting)

(b) the arithmetic root-mean-squared-error (RMS) of existing pointwise uncertainty

values from the original data series

(c) the arithmetic standard-error-of-the-mean (SEM) of calculated detrended values

The DTRNDANL algorithm begins by considering a data sequence of length FWINDOW beginning with the first data point. An arithmetic (*i.e.*, uniformly-weighted) linear-regression detrending is performed on this sub-series data sequence, after which the values of the detrended sub-series sequence are stored internally in memory, in accordance with their corresponding x-values, in either original data space or in SND space.

The algorithm then repeats this process by starting its second pass with the second point in the original time series, its third pass with the third point, and so on, until terminating the detrending process when the filter window requests a sub-series analysis that extends beyond the last point of the original time series data.

All values stored at x-value locations are then averaged to produce the final, detrended time series sequence of values. Uncertainties corresponding to the selection made in (iii) above are then calculated and reported accordingly.

The averaging of sequential sub-series of linear-regression detrended data sequences acts to remove mean non-stationarity (*i.e.*, drift) from the data.

If the data are selected to be reported in SND-space, then variance non-stationarity is also quite aggressively removed.

The selection of an “appropriate” value for a filter window is critical for successful application of this algorithm. For example, to apply DTRNDANL to circadian rhythm data that are recorded in units of hours, a value for FWINDOW of 24-hours would be an appropriate choice (assuming that the dominant rhythm exhibited by the data is near 24-hours in period).

**FFT-NLLS and RAE:**

Data analysis of time series preprocessed by the above mentioned procedure was then performed by FFT-NLLS analysis (Plautz et al., 1977). This algorithm operates such that imported data are first linear regression detrended by FFT-NLLS to produce zero-mean, zero-slope data. An FFT power spectrum is then calculated from the linear regression detrended data. The period, phase, and amplitude of the most powerful spectral peak are used to initialize a one-component cosine function (i.e., an order-1 fit, initially) of the form:

$$y_{\text{LR}}(t)=c+\sum_{j=1}^{M} \alpha_{j}\text{cos}\left[ \frac{2\pi(t+\phi_{j})}{\tau_{j}} \right]$$

in which *y_LR_(t)* is the linear regression detrended time series to which analysis is being performed, *c* is a constant offset term, *M* is the order of fit, *t* is time, and *α_j_*, *ϕ_j_*, *τ_j_* are the amplitude, phase, and period, respectively, of the *j*-th cosine component. The parameters of this function are then estimated by nonlinear least squares minimization by a modified Gauss-Newton NLLS algorithm (Straume et al., 1991; Johnson & Frasier, 1985). Upon convergence, approximate nonlinear asymmetric joint confidence limits are estimated for all parameters (period, phase, amplitude, and constant offset) at 95% confidence probability (Straume et al., 1991). If the amplitude is significantly different from zero, then the procedure is repeated at the next higher order. The two most powerful FFT spectral peaks are then used to initialize a two-component cosine function (i.e., *M* = 2), which is subsequently NLLS minimized to the linear regression detrended data, and confidence limits are again evaluated. This process is repeated iteratively until at least one cosine component is identified with an amplitude that is not statistically significant. Alternatively, the procedure terminates if an error condition is encountered during computation (often an attempt at inversion of a near-singular matrix) or if the relative frequency difference between any two derived cosine components is within 1/*N∆t* (the approximate resolution limit supportable by the data, where *N* represents the number of time points and *∆t* represents the sampling interval). Satisfying any of these termination criteria suggests that a model of too high an order is being attempted to fit the data. Penultimate results are then retained as the most statistically accurate quantitative characterization of the time series. In this way, the period, phase, and amplitude of the data are selected objectively and in fully automated manner with no requirement for user interaction (and, hence, no opportunity for influence of user-introduced bias).

The statistical significance of each derived rhythmic component is assessed by way of the relative amplitude error, RAE, defined as the ratio of, in the numerator, the amplitude error (one-half the difference between the upper minus the lower 95% amplitude confidence limits) to, in the denominator, the most probable derived amplitude magnitude. Theoretically, RAE will range from 0.0 to 1.0; 0.0 indicating a rhythmic component known to infinite precision (*i.e.*, zero error), 1.0 (or greater) indicating a rhythm that is not statistically significant [*i.e.*, error equal to (or exceeding) the most probable amplitude magnitude], and intermediate values indicative of varying degrees of rhythmic determination.

FFT-NLLS is specifically designed to process data sets that are relatively short and/or noisy and is generally capable of extracting relatively weak rhythms. Additionally, it extracts meaningful periods despite moderate mean and variance non-stationarities that may exist in data. It assigns period, phase, amplitude, and rhythmic strength/level of rhythmic determination (the latter via the relative amplitude error, RAE) in a totally automated manner that is free of user-introduced analytical bias.

**Interpretation of FFT-NLLS results:**

The results reported by FFT-NLLS are the best-fit parameter values identified for each data set at the specified level of confidence probability. This last point is important! Reported results will vary depending on the confidence probability level employed during analysis. For valid comparisons of results of analysis of different data files, it is necessary to have identical conditions represented in the results of analysis (*i.e.*, same numbers of data points per data set, same sampling frequency per data set, same confidence probability level used during analysis). The results offered by FFT-NLLS present the amplitude, period, and phase of all statistically significant rhythmic components that the FFT-NLLS algorithm could find at the specified level of confidence probability. Importantly, as such, the parameter error estimates produced by FFT-NLLS are not necessarily estimates of the standard errors of the parameters (unless a confidence probability value of 68.26% was used in analysis). The parameter error estimates produced by FFT-NLLS are estimates of parameter confidence at the user specified level of confidence probability, whether that is 95% or 90% or 99% or 68% or . . . .

**Relative Amplitude Error (RAE) and rhythmic determination:**

The relative amplitude error, RAE, is the value of the amplitude error estimate divided by the value of the most probable amplitude estimate. RAE can range from a value of zero for an infinitely well-determined rhythmic component (zero error) to a value of one, theoretically, for a minimally-determined rhythmic component (error in the amplitude equals the amplitude value itself). There will be times when an RAE value greater than unity will be reported. Although theoretically not possible within the context of linear, symmetric models, the multi-component cosine model that underlies the FFT-NLLS analysis is neither. As such, RAE values greater than unity do sometimes occur (most often when a very-long-period component is identified during analysis to account for long-term drifting that is occurring in the data set being analyzed). In such cases, it is usually a rhythm that is not of interpretive interest, but rather is probably there to accommodate trend.

**Considerations in assessment of phase:**

For purposes of interpretation, any assessment of an analytical determination of a phase angle reference is dependent on at least (i) exactly how time is represented in the data set being analyzed, (ii) any coordinate relationship between the endogenous rhythm and an exogenous periodicity of possible consequence, and (iii) the sign convention employed to represent phase advances versus delays thereof.

Phase angle reference is most commonly considered in terms of maximum, minimum, half-maximum, or half-minimum of some experimental observable.

Representation of time relates to how time zero is referred, because any analytical assessment of phase is implicitly relative to the absolute reference of time zero.

A coordinate relationship between exogenous and endogenous periodicities of consequence refers to the situation in which (for example, in the context of circadian rhythms) one is interested in a phase angle reference on a particular day (*i.e.*, relative to an exactly 24-hour “real-time clock;” the exogenous periodicity of the earth’s rotation, day/night) of some circadian biological rhythm that has been analytically assessed to possess a period of oscillation that is not exactly 24-hours (*i.e.*, a non-24-hour free-running period, which is the endogenous periodicity analytically assessed for biological circadian rhythms).

The sign convention commonly employed in circadian biology refers to advances as positive (*i.e.*, + or >0) and delays as negative (*i.e.*, – or <0). When considering a relative situation comparing two rhythms, the concepts of advance and delay are straightforward. However, when considering an individual rhythm, the reference becomes absolute time zero; thus, in such a case, a positive phase means in advance of time zero (*i.e.*, negative time) and a negative phase means delayed relative to time zero (*i.e.*, positive time). This latter point can be a source of confusion.

**Interpretation of phase:**

In our current procedure, the “phase” value is referenced to whatever time zero is in the data file's time axis. A positive phase value means advanced relative to zero (*i.e.*, negative time) and a negative phase value means delayed relative to zero (*i.e.*, positive time). Please note this relationship in defining phase so as to avoid confusion! The phase marker is cosine acrophase (*i.e.*, peak of cosine wave) and it recurs modulo whatever the period is for that rhythmic component. So, care is necessary when comparing phase reference points so that they are compared correctly with respect to some absolute time marker, not just to what may be a quite-arbitrarily assigned zero-time-value in the time axis of the data files being analyzed. Also, the “phase” referred to is that of a rhythmic component. In the event of multiple rhythmic components being identified in an analysis of a data file, the acrophase of a rhythmic component may not always bear a discernable resemblance to an acrophase of the observed, composite rhythm (*i.e.*, of the composite best-fit curve that may be composed of a linear combination of a number of contributing cosine waves of varying amplitudes, periods, and phase relationships).

**Extrapolation of phase estimates:**

If the time of acrophase of an FFT-NLLS rhythmic component is desired at a time “distant” from absolute time zero of the data set under consideration, it must be calculated relative to its associated period *in modulo*.

This point can hopefully be demonstrated most clearly by some examples. Let us assume in the examples that follow that absolute time zero refers to midnight in “real-clock time.”

1) Suppose an analysis by FFT-NLLS returns a statistically significant period estimate of exactly 24.0 hours, with a corresponding phase estimate reported as -6.0 hours. The time of acrophase between the times of [(–24.0/2)= –12.0] and [(+24.0/2)=12.0] is at *t*=6.0 hours [*i.e.*, –(–6.0)=6.0], thus *ϕ_max,orig_* = 6.0 hours. If one is interested in knowing at what real-clock time the acrophase occurs on the fourth day after time zero as represented in the data set under consideration, the time range of interest becomes 72 ≤ *t* ≤ 96. To accommodate this situation, one must add values of the derived period estimate (*i.e.*, 24.0, in this case) to the time of acrophase (*i.e.*, 6.0, in this case) until a time of acrophase is produced that is within the desired time range (*i.e.*, 72 ≤ *t* ≤ 96, in this case). Since the period estimate in this case is exactly 24.0 hours, the result becomes *ϕ_max,extrap_* = 78.0 hours, which corresponds to *ϕ_max,extrap_* = 6.0 hours relative to the previous midnight.
2) Suppose an analysis by FFT-NLLS returns a statistically significant period estimate of exactly 23.0 hours, with a corresponding phase estimate reported as –6.0 hours. The time of acrophase between the times of [(–23.0/2)= –11.5] and [(+23.0/2)=11.5] is at *t*=6.0 hours [*i.e.*, –(–6.0)=6.0], thus *ϕ_max,orig_* = 6.0 hours. If one is interested in knowing at what real-clock time the acrophase occurs on the fourth day after time zero as represented in the data set under consideration, the time range of interest becomes 72 ≤ *t* ≤ 96. To accommodate this situation, one must add values of the derived period estimate (*i.e.*, 23.0, in this case) to the time of acrophase (*i.e.*, 6.0, in this case) until a time of acrophase is produced that is within the desired time range (*i.e.*, 72 ≤ *t* ≤ 96, in this case). Since the period estimate in this case is exactly 23.0 hours, the result becomes *ϕ_max,extrap_* = 75.0 hours, which corresponds to *ϕ_max,extrap_* = 3.0 hours relative to the previous midnight.
3) Suppose an analysis by FFT-NLLS returns a statistically significant period estimate of exactly 25.0 hours, with a corresponding phase estimate reported as –6.0 hours. The time of acrophase between the times of [(–25.0/2)= –12.5] and [(+25.0/2)=12.5] is at *t*=6.0 hours [*i.e.*, –(–6.0)=6.0], thus *ϕ_max,orig_* = 6.0 hours. If one is interested in knowing at what real-clock time the acrophase occurs on the fourth day after time zero as represented in the data set under consideration, the time range of interest becomes 72 ≤ *t* ≤ 96. To accommodate this situation, one must add values of the derived period estimate (*i.e.*, 25.0, in this case) to the time of acrophase (*i.e.*, 6.0, in this case) until a time of acrophase is produced that is within the desired time range (*i.e.*, 72 ≤ *t* ≤ 96, in this case). Since the period estimate in this case is exactly 25.0 hours, the result becomes *ϕ_max,extrap_* = 81.0 hours, which corresponds to *ϕ_max,extrap_* = 9.0 hours relative to the previous midnight.

Exactly the same process will apply regardless of the sign of the phase. *I.e.*, if phase is positive, then its negation becomes negative, indicating that acrophase occurred in the negative time domain between the times of (–*τ*/2) and (*τ*/2) (*i.e.*, *ϕ_max,orig_* < 0). Then, if a phase optimum is desired on some day other than the day nearest *t*=0, the relevant range of times must be determined after which exactly the same extrapolation procedure is employed, modulo *τ*. Of course, if the relevant range of times is prior to *t*=0 instead of after *t*=0, then the time of phase optimum is arrived at by subtraction, rather than addition, modulo *τ*.

So, the key steps to consider are to (i) negate the phase value reported by FFT-NLLS, (ii) define the range of times between which a phase optimum estimate is desired, and (iii) extrapolate modulo *τ* by period addition (forward in time relative to *t*=0) or period subtraction (backward in time relative to *t*=0) until an acceptable phase value is produced (after which it may be again converted into relative real-clock time, for example).

**Description of the software analysis package included with the LumiCycle:**

*Baseline fit:* Baseline drift correction is accomplished by applying a polynomial-fit of user-specified order to the data series.

*Period calculation:* Baseline-corrected data may have their oscillatory period estimated by five available methods.

(i) Sin fit identifies the largest sinusoidal component in the data by calculating a power spectrum by the following equation as a function of a range of closely spaced frequencies:

$$P(f)=\left( \sum_{i=1}^{N} Y(t_{i})\text{sin}(2\pi\text{ft}_{i}) \right)^{2}+\left( \sum_{i=1}^{N} Y(t_{i})\text{cos}(2\pi\text{ft}_{i}) \right)^{2}$$

The power, *P(f)*, is calculated by performing the summations at each frequency, *f*, over all *N* data points comprising the baseline-corrected data series, *Y(t)*, at each of the corresponding time points, *t_i_*. The period (1/*f_peak_*) corresponding to the peak power is selected as the dominant period, after which the associated optimal phase is calculated.

(ii) Sin fit (damped) builds upon the results of the previous <Sin fit> analysis by additionally accounting for damping of oscillatory amplitude according to an exponential decay function. The exponential decay is fit to the baseline-corrected time-points of the original data series occurring at the times corresponding to the peaks and troughs of the best-fit <Sin fit> curve.

(iii) Sin fit (undamp) is a further extension of the results of the previous <Sin fit (damped)> analysis in that the data of the baseline-corrected series are divided by the best-fit exponential derived from the <Sin fit (damped)> analysis.

(iv) LM fit performs a simultaneous Levenberg-Marquardt least-squares parameter optimization of the following equation to the baseline-corrected series data:

$$Y(t)=A\text{sin}(2\pi\text{ft}+\phi)\text{exp}(-t/\tau)+C$$

In this case, the parameters *A*, *f*, *ϕ*, *τ*, and *C* are least-squares optimized to the baseline-corrected series data, *Y(t)*.

(v) Chi-squared periodogram calculations may also be performed, according the method of Sokolov & Bushell.

*Goodness-of-fit:* Goodness-of-fit is characterized by the fractional variance in the baseline-corrected data series that is accounted for by the corresponding best-fit analytical result.

We thank Dr. David Ferster (Actimetrics) for the information above relating to the software analysis package presently included with the LumiCycle and for permission to include it in our Supporting Information.

**References for Supporting Information**:

Johnson ML, Frasier SG (1985) Nonlinear least squares analysis. *Methods Enzymol* 117: 301-342.

Plautz JD, Straume M, Stanewsky R, Jamison CF, Brandes C, Dowse HB, Hall JC, Kay SA (1997) Quantitative analysis of Drosophila period gene transcription in living animals. *J Biol Rhythms* 12: 204-217.

Straume M, Frasier-Cadoret SG, Johnson ML (1991) Least squares analysis of fluorescence data. *In* Topics in Fluorescence Spectroscopy, Volume 2: Principles, JR Lakowicz, ed, pp. 117-240. Plenum, New York.

Sokolove PG, Bushell WN (1978) The chi-square periodogram: Its utility for analysis of circadian rhythms. *J Theor Biol* 74: 131-160.

*τ* = period

*ϕ* = phase
